# Structure Prediction and Validation of the ERK8 Kinase Domain

**DOI:** 10.1371/journal.pone.0052011

**Published:** 2013-01-11

**Authors:** Angela Strambi, Mattia Mori, Matteo Rossi, David Colecchia, Fabrizio Manetti, Francesca Carlomagno, Maurizio Botta, Mario Chiariello

**Affiliations:** 1 Istituto Toscano Tumori-Core Research Laboratory, Signal Transduction Unit, AOU Senese, Siena, Italy; 2 Istituto di Fisiologia Clinica, Consiglio Nazionale delle Ricerche (CNR), Siena, Italy; 3 Dipartimento Farmaco Chimico Tecnologico, Università degli Studi di Siena, Siena, Italy; 4 Università degli Studi di Siena, Siena, Italy; 5 Dipartimento di Biologia e Patologia Cellulare e Molecolare, Università degli Studi di Napoli, Napoli, Italy; German Research School for Simulation Science, Germany

## Abstract

Extracellular signal-regulated kinase 8 (ERK8) has been already implicated in cell transformation and in the protection of genomic integrity and, therefore, proposed as a novel potential therapeutic target for cancer. In the absence of a crystal structure, we developed a three-dimensional model for its kinase domain. To validate our model we applied a structure-based virtual screening protocol consisting of pharmacophore screening and molecular docking. Experimental characterization of the hit compounds confirmed that a high percentage of the identified scaffolds was able to inhibit ERK8. We also confirmed an ATP competitive mechanism of action for the two best-performing molecules. Ultimately, we identified an ERK8 drug-resistant “gatekeeper” mutant that corroborated the predicted molecular binding mode, confirming the reliability of the generated structure. We expect that our model will be a valuable tool for the development of specific ERK8 kinase inhibitors.

## Introduction

The ease and the rate of genome sequencing are higher today than ever before. Conversely, experimental techniques for protein structure determination are characterized by a much slower rate, entailing that three-dimensional (3D) structure for many potential drug targets will likely be not experimentally solved but predicted instead. For this reason, modeled structures obtained by computational techniques, once validated, will represent an irreplaceable reservoir for modern drug design and development. In this context, in the last 10–15 years, protein kinases have become particularly interesting drug targets for pharmaceutical industry. In cancer research only, over 50% of the current projects are indeed estimated to focus on kinase targets [1].

There are around 500 members of the protein kinase superfamily encoded by the human genome, whose degree of similarity in the catalytic domain poses many challenges to develop really specific inhibitors targeting the ATP cavity [2]. Still, this similarity is the property that can be also exploited for structural modeling. In turn, such 3D knowledge will be important to predict sensitivity to ATP competitive inhibitors and represents the rationale for the development of more specific compounds (not only type I inhibitors, but also type II inhibitors and type III or allosteric inhibitors) [3]. Importantly, the predictive value of a reliable 3D structure will be also a useful tool to rationally modulate a possible second-line therapy when resistance arises.

Mitogen-activated protein kinases (MAPKs) regulate evolutionarily conserved signaling pathways affecting all essential cellular functions. For this reason, abnormalities in MAPKs signaling also play a critical role in the development and progression of cancer [4]. Extracellular signal-regulated kinase 8 (ERK8, MAPK15) is the last identified member of the MAPK family [5]. It is a proline-directed serine/threonine kinase featuring the distinctive Thr-Xaa-Tyr (TXY) motif in the activation loop [6], whose post-translational modifications appears to be performed through autophosphorylation [7]. Still, its activity can be further modulated by serum, DNA-damage and human oncogenes [5], [8], [9]. Importantly, ERK8 has been implicated in cell transformation [10], in the protection of genomic integrity [11], and has been described as a potent regulator of telomerase activity [12] and of the autophagic process [13]. Consequently, it has been proposed as a novel therapeutic target for cancer. Ultimately, ERK8 has been also reported to stimulate the activity of the *JUN* proto-oncogene [10] and to reduce the activity of different nuclear receptors [14], [15]. Specific ERK8 inhibitors would thus represent useful tools for the study of its still poorly characterized signaling pathways and could confirm the clinical potential of ERK8 targeting for cancer therapy.

With the aim of developing a 3D structure of ERK8, we took advantage of the similarity of its ATP-binding domain to other MAPKs for structural modeling. Once obtained, we successfully confirmed the reliability of our model by applying a structure-based virtual screening protocol that allowed us to identify molecular scaffolds able to inhibit ERK8 kinase activity. Importantly, we confirmed the binding of such molecules to the ERK8 ATP binding pocket both by ATP competition assays and by using the first reported ERK8 drug-resistant “gatekeeper” mutant. Overall, our experimental data clearly sustain the predictive ability of the generated model for the ERK8 kinase domain and promise its utility in a drug-design perspective.

## Materials and Methods

### Homology Modeling

All the primary sequences were obtained from UniPROT protein sequence database [16]. Sequence similarity searches were carried out using BlastP [17]. Based on previous homology modeling studies on protein kinases [18], sequence alignment was performed by CLUSTAL W [19] with a gap open penalty of 10 and a gap extension penalty of 0.05. Also other parameters were kept at their default values. The alignment was also tested with the standard protocol of the T-Coffee method [20] (Fig. S1). The crystal structure of FUS3, ERK2, p38α and CDK2 were from the Protein Data Bank [21]; entries ID: 2B9F [22], 1ERK [23], 1P38 [24], 1HCK [25].

The kinase domain of ERK8 (residues 12-345) was obtained using Modeller 9v5 package [26]. The best protein model was chosen on the basis of the DOPE (Discrete Optimized Protein Energy) assessment method as implemented in Modeller. Cartoons were prepared with Pymol software (The PyMOL Molecular Graphics System, Version 1.2r3pre, Schrödinger, LLC).

### Molecular Dynamics simulations

The AMBER10 program [27] was used for relaxing protein coordinates by means of energy minimization and molecular dynamics simulations. The ff99 and the GAFF force fields were used for protein and organic ligands, respectively. A rectilinear box of TIP3P water molecules buffering 12 Å was added to solvate the Mg^++^ ERK8/ADP complex, while Cl^−^ counterions were added to neutralize the system. Energy minimization was performed by using a combination of the steepest descent and the conjugate gradient algorithms. First, the solvent was energy minimized while keeping solute's coordinates frozen. Then, the solute alone was energy minimized prior to relax the whole solvated complex. The system was then heated to 300 K for 100 ps and the density was equilibrated for 100 ps before producing MD trajectories. The SHAKE algorithm was used to restrain bonds involving H atoms. A time-step of 1 fs was used. The unbound approach for Mg^++^ ions was applied. Mg^++^ and ADP were restrained by a force constant decreasing from 25 to 0 kcal/mol·Å^2^ during the first 1 ns of MD simulation. Unrestrained trajectories were produced for 1.5 ns (Fig. S3).

### Pharmacophores

Ligand-based pharmacophore models were generated by using the “Common Feature Pharmacophore Generation” protocol of Discovery Studio 2.5 (Discovery Studio, v2.5. San Diego: Accelrys Software Inc., 2009) on a training set of molecules known to inhibit ERK8 [28]. With this method, two different pharmacophore models were generated, composed of 4 and 5 features, respectively.

Structure-based pharmacophore models were generated by following two different approaches: 1) the Ligandscout 3.0 software [29] was used to derive a pharmacophore model accounting for the interactions performed by ADP toward Mg^++^ loaded ERK8, starting from the representative structure obtained by MD simulations (the refined ERK8 structure). Features accounting for the interaction of phosphate groups with the Mg ion were manually removed; 2) the GRID software (GRID 22; Molecular Discovery Ltd.) [30] was used to explore possible molecular determinants involved in ligand binding within the ERK8 active site, as generated by MD simulation. Starting from the representative MD structure, coordinates of ADP and Mg^++^ were removed. Selected probe atoms were: O (sp2 carbonyl oxygen), DRY (the hydrophobic core), C3 (methyl CH3 group), C1 = (sp2 CH aromatic or vinyl), N1 (neutral flat NH, e.g. amide), O1 (alkyl hydroxyl OH group), N: (sp3 N with lone pair). Points of energy minimum were converted into pharmacophoric features according to the GBPM approach [31].

### Molecular Docking

Docking calculations were performed by using the GOLD program, version 4.1.2 [32]. The Chemscore scoring function [33], [34] was used, as it is able to finely reproduce the binding mode of ADP described in the refined ERK8 structure. Default parameters of the Genetic Algorithm (GA) were used, whereas the efficiency was set at 150%. Twenty runs for each ligand were generated by the GA, allowing the early termination after 5 consecutive runs having a root mean square deviation (rmsd) below the tolerance limit of 1.5 Å. The binding site was centered on the side chain of leucine 144 and had a radius of 16 Å.

### Expression Vectors

Bacterial expression vector pGEX4T3-ERK8 was generated by subcloning the ERK8 cDNA, obtained by restriction enzyme digestion from the already described pCEFL-HA-ERK8 [8], into the pGEX4T3 vector in frame with the GST tag. ERK8 gatekeeper mutants were generated using the QuikChange site-directed mutagenesis kit (Stratagene), using pGEX4T3-ERK8 as a template. pGEX4T3-ERK8 kinase dead (KD) was generated subcloning ERK8 K42R cDNA (kindly provided by M. Abe) into the pGEX4T3 vector. The identity and integrity of all the vectors were confirmed by DNA sequencing.

### Bacterial expression of GST-fusion proteins

The BL21 Lys strain of *Escherichia coli* (*E. coli*) was transformed with the pGEX4T3 vectors encoding for the full-length form of ERK8 protein and of the different mutants. Bacterially expressed GST-fusion proteins were purified as previously described [35]. The baculovirus-expressed GST-fusion ERK8 protein is from Carna Biosciences, Inc.

### Reagents

Ro-318220 is from Calbiochem; radiolabeled [γ-^32^P] ATP is from PerkinElmer; screened compounds are from Asinex.

### In vitro Kinase Assay

Purified GST-fusion protein (50 ng/sample) was incubated 30 min at 30°C in kinase buffer [25 mM HEPES (pH 7.6), 0.1 mM Na_3_VO_4_, 20 mM β-glycerophosphate, 2 mM DTT, 20 mM MgCl_2_] with 5 µg/sample of MBP (Sigma) as generic substrate, 2.5 µCi [γ-^32^P]ATP and unlabeled ATP (final concentration 5 µM) [28], unless differently indicated. Candidate compounds dissolved in dimethyl sulfoxide (DMSO) were added as needed (an equal volume of DMSO was added to control samples). Reaction was stopped adding 5X Laemmli buffer and resolved by SDS-PAGE. Dried gels were then exposed to phosphorimager (Typhoon 8600, Molecular Dynamics) and ^32^P incorporation on MBP was estimated by densitometry (ImageQuant TL Software, GE Healthcare).

Alternatively, for samples to be analyzed by liquid scintillation counter, reaction mixture was spotted (after incubation at 30°C for 30 minutes in the same kinase buffer described above) on Whatman (P81) 3MM paper and immediately immersed in 1% phosphoric acid to terminate the reaction [36]. After four washes with fresh phosphoric acid, scintillant solution was added (BioFluor, PerkinElmer) and ^32^P incorporation of triplicates was measured with a β-counter scintillator (TRI-CARB 2000, Packard). All the assays were replicated twice and the means of the replicates were calculated. For Coomassie staining, before adding radiolabeled ATP, an aliquot of each sample prepared for kinase assay was loaded on SDS-PAGE gel, stained with SimplyBlue SafeStain (Invitrogen), and revealed using the Odyssey Infrared Imaging System (Li-Cor Biosciences).

### Western Blots

Proteins in storage buffer (50 mM Tris pH 8.0, 0.5 mM DTT, 0,5 mM EDTA and 10% glycerol) were loaded on SDS-PAGE gel, transferred to Immobilon-P PVDF membrane (Millipore), probed with anti-ERK8 primary antibody (custom preparation) and secondary antibody HRP-conjugated anti-rabbit IgGs (Santa Cruz Biotechnology), and revealed by enhanced chemo-luminescence detection (ECL Plus; GE Healthcare).

## Results

### Homology Modeling

**Figure 1:**
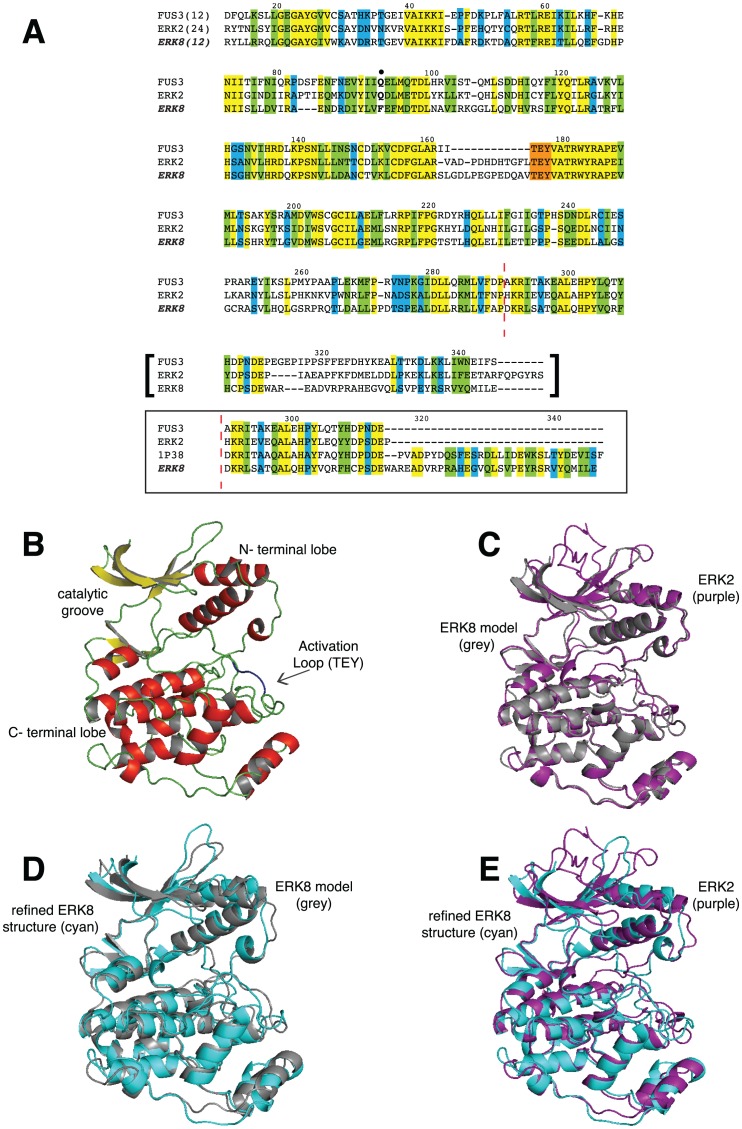
ERK8 kinase domain model. (A), Multiple sequence alignment between ERK8 and the selected templates FUS3 and ERK2. Numbering is referred to human ERK8 cDNA sequence as defined in Uniprot accession number Q8TD08. Consensus code: “yellow” indicates positions which have a single, fully conserved residue; “green” indicates conservation between groups of strongly similar properties; “blue” indicates conservation between groups of weakly similar properties. Gatekeeper residue is in bold and indicated by a full black circle. The TEY activation motif is in red (activation loop spans from the DFG motif to the APE motif, residues 155–187). The region in square brackets has been substituted (starting from the position indicated with the dashed red line) with the alignment highlighted in the bottom square that includes p38α. (B), Model of the ERK8 kinase domain (residues 12–345 of the full-length 1–544 protein) obtained by means of homology modeling protocol. Conserved kinase domain features are indicated, β-sheets colored in yellow, α-helices colored in red, loops colored in green, TEY activation motif colored in blue. (C), Superimposition of the same ERK8 model (grey) with the ERK2 template (purple). (D), Evolution of ERK8 structure with the MD refinement. Superimposition of the ERK8 model (grey), used as MD input, with the representative final structure (the refined ERK8 structure) (cyan) obtained after the simulation. (E), Superimposition of the refined ERK8 model (cyan) with the ERK2 template (purple).

ERK8 is a 544-amino acid protein with a typical MAPK catalytic domain and a peculiarly long C-terminal extension [5]. The degree of conservation of the catalytic domain, as compared with other MAPKs, is adequate to predict its 3D structure by means of homology modeling procedure, whereas the C-terminal region does not share a significant sequence identity with any known protein, thus impeding any attempt of modeling the whole protein structure with this approach. Therefore, we generated a 3D structure of the ERK8 kinase domain by homology modeling procedure, in the perspective of obtaining a reliable model to next screen for novel ERK8-directed ATP competitive scaffolds. We selected FUS3 (*S. cerevisiae* MAPK) and ERK2 X-ray structures as templates, featuring a percentage of sequence identity with ERK8 kinase domain of 45% and 44%, respectively (similarity is 64% and 62%, respectively) (Fig. 1A). This degree of homology guaranteed a high-quality model structure [37]. In this context, while we used ClustalW as our leading program to align all the sequences, in order to get a higher confidence on the ability to correctly align motifs and domains, we also tested the multiple sequence alignment with T-Coffee (Fig. S1), another widely used method. As expected, we obtained almost complete identity of the two final alignments in the core catalytic unit.

While featuring high homology with most of FUS3 and ERK2 kinase domains, the last thirty residues of ERK8 kinase domain did not share such high homology with the selected proteins and also provided lower level of agreement between the two methods of alignment (Fig. 1A and Fig. S1). Therefore, we decided to include a third template, namely p38α, which shares higher homology with ERK8 in this particular segment obtaining the final alignment reported in Figure 1A. Based on this alignment, we next performed homology modeling using Modeller 9v5 [26]. Out of the twenty models that we generated, the top five showed a backbone rmsd lower than 1.5 Å. Accordingly, the model having Mg^++^ and ADP within the catalytic active site, endowed with the lower DOPE score was selected as our ERK8 model. The structure showed the overall MAPK topology with an N-terminal and a C-terminal lobe forming the catalytic groove (Fig. 1B). Superimposition of the modeled ERK8 structure with corresponding atoms of ERK2 (Fig. 1C) and FUS3 (Fig. S2) templates resulted in a rmsd for Cα atoms of <1 and 1.2 Å, respectively. This model was further refined by means of standard *in silico* procedures to optimize protein-ligand complex structure for ligand design approaches [38].

### Molecular Dynamics Simulation

The structure of ERK8 generated by homology modeling was relaxed by means of a Molecular Dynamics (MD) simulation. With respect to our previous approach to protein kinases study [18], where the homology model was relaxed by means of energy minimization, here we further used MD simulations to better account for protein flexibility [39]. In detail, the system was first solvated and neutralized by the addition of counterions to resemble a physiological situation. Next, energy minimization was performed to solve bad contacts prior to heating and equilibrating the system, and eventually generating unrestrained MD trajectories for 1.5 ns. As expected for kinase proteins in complex with a ligand, the system was highly stable during the time of simulation [18], [39], [40], especially within the ATP binding site (Fig. S3). Therefore, we considered 1.5 ns a sufficient amount of time to refine this protein structure before performing a structure-based virtual screening. The average structure was calculated, whereas the frame with the lowest rmsd with respect to the average structure was considered representative for the system, and used for structural considerations, as well as for further studies (Fig. 1D). Psi and Phi angles of protein residues, taken from the representative structure, were plotted over a Ramachandran map showing that 99% residues were in the most favorable or additional allowed regions, whereas only three residues (1%) were in disallowed regions. However, these latter residues were significantly far from the ATP binding site. The representative structure resulting from MD simulations (hereafter referred to as refined ERK8), that showed a <2 Å rmsd for Cα atoms in comparison to corresponding atoms in ERK2 (Fig. 1E), was first validated by self-docking ADP within the catalytic site by using the GOLD docking program (see below). The docking-based binding pose of ADP was very similar (rmsd <1.5 Å) to that found in the refined ERK8, and to the crystallographic ligand pose within the active site of the template FUS3 (Fig. S2) [22].

### Structure-based pharmacophores and molecular docking

**Figure 2:**
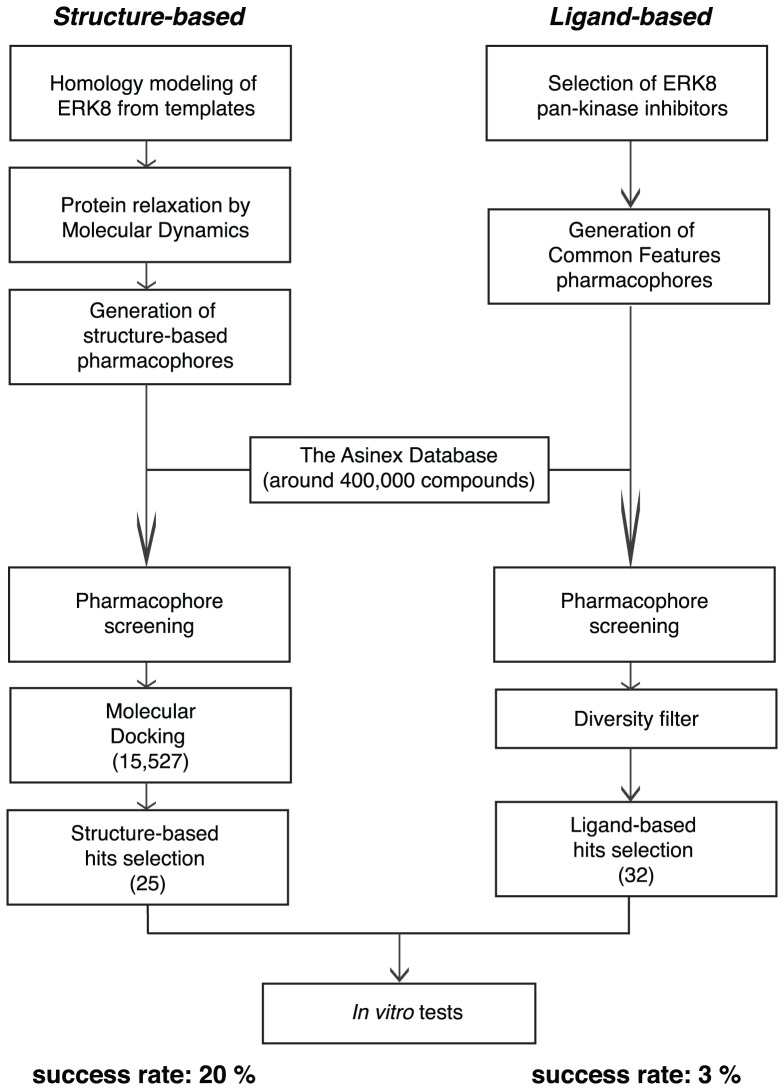
Flowchart of the *in silico* protocol. Computational steps applied to select all the hit compounds to be tested *in vitro*. In each set the percentage of success rate refers to the ratio between the number of active molecules and the number of tested molecules in the following experimental screening: purified GST-ERK8 protein (50 ng/sample) was used in kinase assays. Candidate compounds were dissolved in dimethyl sulfoxide (DMSO) and tested at fixed concentration of 50 µM (an equal volume of DMSO was added to control samples). Reactions were resolved by SDS-PAGE and ^32^P incorporation on MBP was estimated by densitometry. Molecules were classified as active when the residual kinase activity was less than 50% in comparison to control samples.

**Figure 3:**
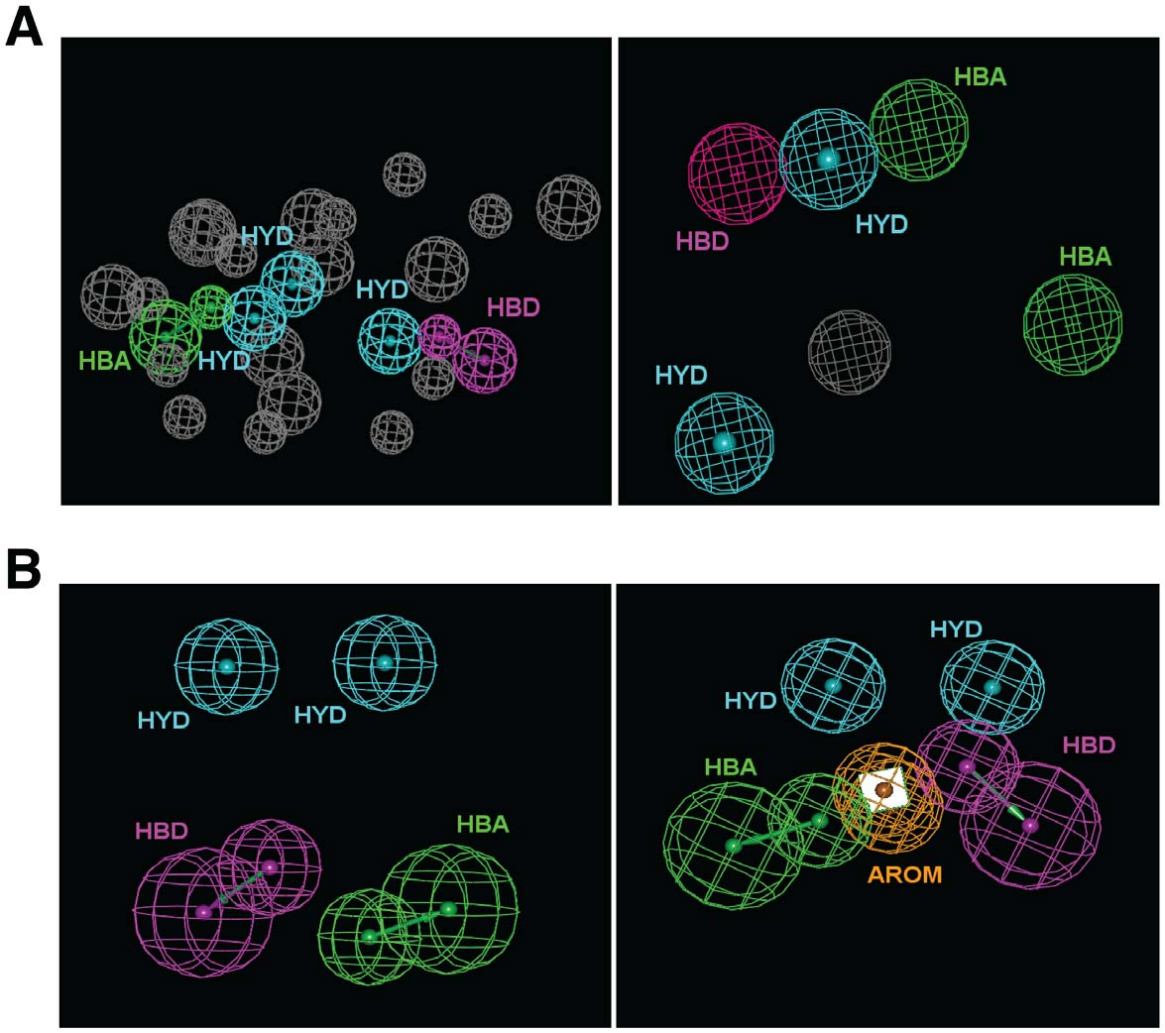
Pharmacophore models. (A), Left panel, Structure-based pharmacophore generated from the Mg^++^ loaded ERK8/ADP complex (coordinates were taken from the refined ERK8 structure) by using the Ligandscout software. Right panel, Structure-based pharmacophore generated by the GRID-based pharmacophore modeling approach, starting from the ligand-bound refined structure of ERK8. Features code: HYD = hydrophobic; HBA = H-bond acceptor; HBD = H-bond donor; AROM = aromatic ring; grey spheres are excluded volumes. (B), The two ligand-based pharmacophores generated with the training set of 18 different inhibitors active towards ERK8 (from Bain J, et al., 2007). Features code same as above.

Pharmacophore models represent a useful tool to filter large compound libraries on the basis of steric and electronic requirements. In this virtual screening campaign, the generation of structure-based pharmacophores was allowed by the availability of the refined ERK8 structure generated by homology modeling and MD, as reported above (Fig. 2). Pharmacophores were built by using two different strategies: one – the Ligandscout software [29] was used to transform the ADP interaction pattern within the catalytic site of the refined ERK8 strucure into a pharmacophore model, which was composed of three hydrophobic, one H-bond acceptor, one H-bond donor and 19 exclude volume features (Fig. 3A, left panel). Two – the GRID-based pharmacophore modeling approach (GBPM) [30], [31] was applied to generate a pharmacophore model which accounted for the space regions endowed with the lowest interaction energy for representative probe atoms within the catalytic site of ERK8 (Fig. 3A, right panel). This model was composed of two hydrophobic, one H-bond donor, two H-bond acceptor and one excluded volume features. After format conversion, necessary to allow compatibility with Discovery Studio (Accelrys), pharmacophore models were used as 3D queries to filter the Asinex library of compounds (about 400,000 small molecules) using a procedure previously described [41]. The 15,527 compounds that survived to the structure-based pharmacophore filtration were submitted to the following docking step.

Indeed, we reckoned that the ability to identify ATP competitive compounds by molecular docking, relying on the kinase domain model of ERK8, might represent a good approach to corroborate the structure itself. Accordingly, the refined ERK8 structure was used as a rigid receptor in a docking-based hit identification procedure. The GOLD program (version 4.1.2) [32] was used with the Chemscore scoring function [33], [34]. Docked compounds were sorted on the basis of their score and the three top-ranking poses of the first 250 molecules were visually inspected within the catalytic active site of ERK8. Molecules showing a clear overlapping between the three poses (rmsd <1.0 Å) were deemed top priority. Based on the predicted binding mode as well as on chemical diversity criteria, the most promising 25 virtual hits were selected for further experimental investigation (Fig. 2).

### Ligand-based pharmacophores

Beside the structure-based virtual screening approach, a ligand-based strategy for hit identification was also attempted, based on the activity data of 18 pan-kinase inhibitors (Fig. S4) toward ERK8 [28]. In general, although results of structure- and ligand-based approaches are not superimposable, combination of different strategies could enhance the probability to identify chemically diverse molecular scaffolds, especially when performing the first round of a computer-aided drug design approach toward a given target system.

Two ligand-based common feature pharmacophore models were generated by using Discovery Studio 2.5, starting from known ERK8 inhibitors [28]. The first model was composed of two hydrophobic, one H-bond acceptor and one H-bond donor features, whereas the second model was composed of two hydrophobic, one aromatic ring, one H-bond donor and one H-bond acceptor features (Fig. 3B). Notably, these pharmacophores are significantly different from each other and from structure-based models. Ligand-based pharmacophores were used as 3D queries to filter the Asinex database, the resulting compounds were inspected for chemical diversity, and the selected 32 putative hits were submitted to experimental investigation (Fig. 2).

### Experimental Screening

To assess the effect of *in silico*-selected compounds on ERK8 catalytic activity, we generated an N-terminal, GST-tagged form of the full-length protein in *E. coli*. In a classical kinase assay with radiolabeled ATP, bacterially expressed GST-ERK8 was found to be constitutively active (data not shown), as previously described [7]. We, therefore, monitored ERK8 ability to phosphorylate a typical substrate, Myelin Basic Protein (MBP) [28] in the presence of each candidate compound at a fixed concentration of 50 µM. As a positive control of ERK8 catalytic inhibition, we used the ATP competitive inhibitor Ro-318220. This molecule, originally developed as a protein kinase C (PKC) inhibitor [42], has been already proven to potently inhibit ERK8 [7], [12], [13]. By this approach, we tested the 32 compounds obtained from the ligand-based virtual screening and the 25 coming from the structure-based virtual screening. In order to classify the analyzed compounds in a binary fashion, we set a threshold at 50% of residual kinase activity with respect to control samples containing no inhibitors: molecules capable of an effect equal or higher than the threshold were labeled as “active”, “not active” otherwise. The structure-based derived subset was populated by a significant percentage (20%) of “active” molecules as compared to the ligand-based approach that showed only 3% of success rate (Fig. 2). Interestingly, besides the “active” molecules, a high percentage of the total tested compounds showed at least a partial ability to decrease ERK8 activity (Table S1). This finding, therefore, indicates that the modeled 3D kinase domain of the refined ERK8 has a reliable structure and that, in this specific case, the structure-based approach is to be preferred over the ligand-based one to identify molecular scaffolds potentially interesting for further optimization.

### In vitro characterization of selected scaffolds

**Figure 4:**
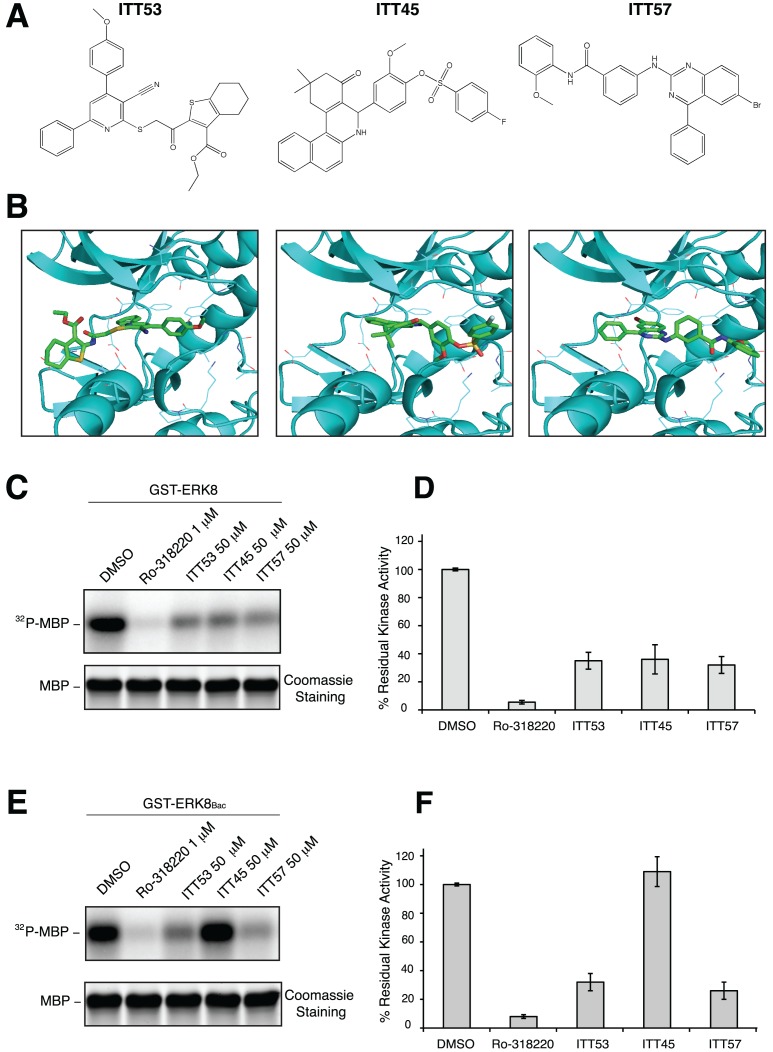
Effect of selected molecular scaffolds on bacterial and eukaryotic GST-ERK8. (A), Molecular structure of selected compounds. (B), Binding mode of each compound as obtained after the molecular docking step. The ITT molecules are showed as sticks and colored by atom type. ERK8 protein structure is represented by secondary structure cyan elements. (C), Samples of GST-ERK8 from *E. coli* with the indicated concentration of inhibitors were subjected to kinase assay. Reactions were resolved by SDS-PAGE and ^32^P incorporation on MBP was estimated by densitometry (upper panel). Coomassie staining verified that equal amounts of substrate were loaded (lower panel). (D), The average results of three independent experiments done in duplicate ± SD are plotted. (E), Samples of GST-ERK8_Bac_ with the indicated concentration of inhibitors were subjected to kinase assay. Reactions were resolved by SDS-PAGE and ^32^P incorporation on MBP was estimated by densitometry (upper panel). Coomassie staining verified that equal amounts of substrate were loaded (lower panel). (F), The average results of three independent experiments done in duplicate ± SD are plotted.

ITT45, ITT53 and ITT57 (Fig. 4A–B and Table S1), the most potent identified inhibitors of ERK8 catalytic activity, were selected among “active” molecules also considering their significant chemical diversity. Indeed, the Tanimoto similarity indexes calculated by using ECFP_6, MDLPublicKeys and FCFP_6 sets of fingerprints were below 0.1, 0.5 and 0.2 each other, respectively. Moreover, to the best of our knowledge, this is the first time that these molecular scaffolds are proposed as ERK inhibitors. The three compounds were submitted to an in-depth *in vitro* characterization aimed at fully validating the refined ERK8 structure and, consequently, the overall computational protocol. For this purpose, we confirmed the inhibitory effect of the selected compounds, as found during the preliminary screening, showing a calculated percentage of residual kinase activity of 36±7%, 35±8% and 32±5% for ITT53, ITT45 and ITT57, respectively (Fig. 4C and Fig. 4D).

**Figure 5:**
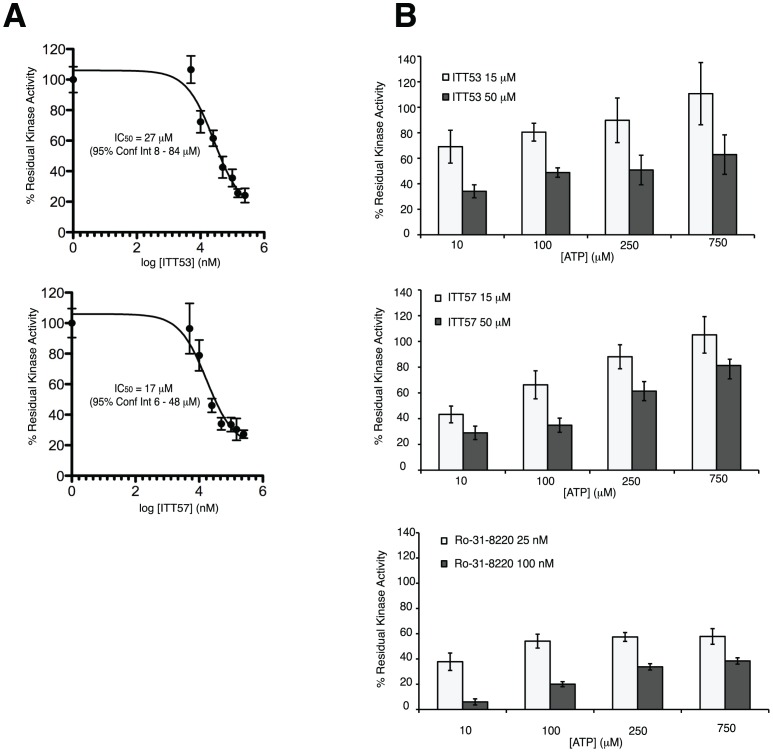
*In vitro* characterization. (A), Dose/response curves for ITT53 and ITT57 on GST-ERK8_Bac_. Results are reported as residual MBP phosphorylation levels compared with the control (DMSO). The average results of two independent experiments done in triplicate ± SD are plotted with the curve-fitting PRISM software (GraphPad). The concentration of drug that inhibited activity by 50% (IC_50_) is shown. (B), ITT53, ITT57 and Ro-318220 ATP competition assay on GST-ERK8_Bac_. Inhibition values are reported as percentage of residual MBP phosphorylation levels (i.e., residual kinase activity) compared with the control (DMSO). Results for the two indicated concentrations of ITT53, ITT57 and Ro-318220 (top, middle, bottom panel, respectively) at four different ATP doses were plotted. The average results of two independent experiments done in triplicate ± SD are plotted.

Although widely used as a source of protein kinases for studying their activity and response to inhibitors [28], it is well established that *E. coli*-based bacterial expression systems do not necessarily ensure a correct post-translational processing of heterologously expressed proteins, often required for fully and correctly controlled activity. In this regard, the baculovirus-based insect expression system has been used to produce recombinant proteins, because insect cells can perform correct post-translational modification of heterologous proteins [43]. Based on this information, we next decided to test our compounds on GST-ERK8 produced from baculovirus-infected insect cells. GST-ERK8 purified through this system (hereafter named GST-ERK8_Bac_) was then used in classical kinase assays to test the three scaffolds. ITT53 and ITT57 efficacy was almost equivalent to that observed with bacterially expressed protein (i.e., 32±6% and 26±6% of residual kinase activity, respectively), whereas, surprisingly, no effect was obtained by using ITT45 (Fig. 4E and Fig. 4F). This observation showed the importance of confirming the results obtained from bacterially-produced ERK8 also by using an eukaryotic expression system and also suggested a potentially specific role for post-translational modifications in the regulation of ERK8 activity. Based on this evidence, we decided to perform additional characterization only on ITT53 and ITT57, which gave best chances to affect fully active ERK8. Dose/response curves were then carried out on GST-ERK8_Bac_ and estimated half-maximal inhibitory concentration (IC_50_) values for ITT53 and ITT57 were 27 µM (95% confidence interval 8–84 µM) and 17 µM (95% confidence interval 6–48 µM), respectively (Fig. 5A). Next, we tested ITT53 and ITT57 in a competition assay with different concentrations of ATP. As reported in Figure 5B, increasing ATP concentrations induced a decrease in the inhibitory action of ITT53 and ITT57. This profile was compatible with a competitive mechanism with the natural substrate, and indirectly supported the reliability of our ERK8 kinase domain model that has been used to select these ATP binding pocket small molecule inhibitors. As an additional control, the ATP competition assay profile of ITT53 and ITT57 displayed the same characteristics of the positive control Ro-318220 (Fig. 5B), already described as an ATP competitive kinase inhibitor [42]. Altogether, these results indicate that the two molecules are able to inhibit the kinase activity of the full-length protein expressed both in *E. coli* and in a eukaryotic system. The IC_50_ values represent a valuable starting point for molecules obtained from a first “*in silico*-*in vitro*” screening process. They also show a behavior that is compatible with a competition mechanism with the natural substrate ATP.

### Binding Mode Analysis

**Figure 6:**
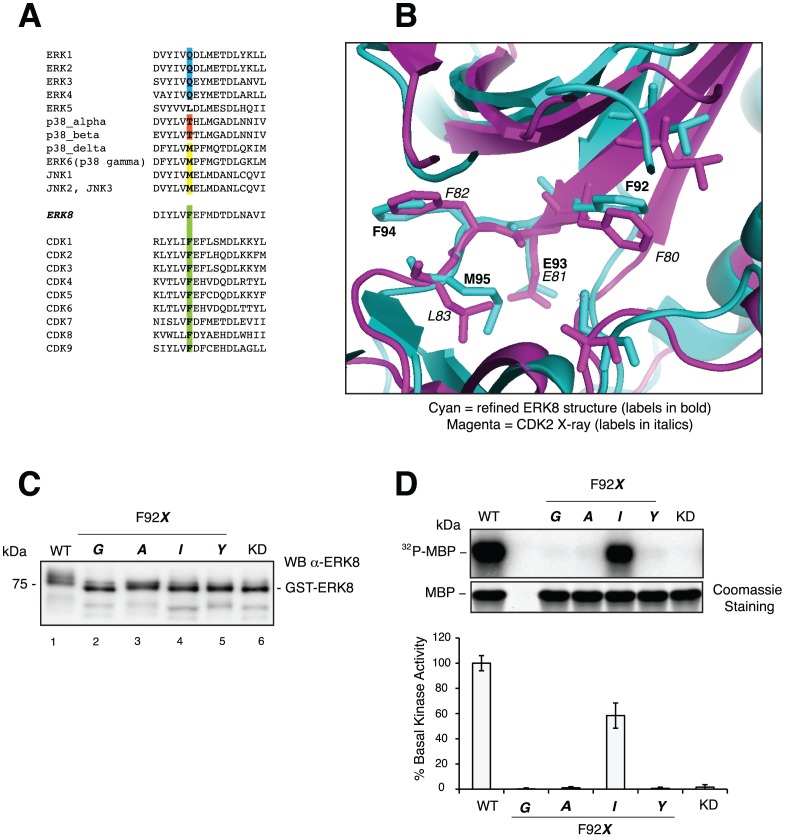
Gatekeeper mutants. (A), Multiple sequence alignment of gatekeeper region among different members of the MAPK and CDK families of kinases. The position corresponding to the gatekeeper residue is highlighted. (B), Superimposition of the refined ERK8 structure (cyan) and CDK2 (magenta) X-ray structure. (C), Western Blot control of GST-fusion proteins from *E. coli*. Each lane was loaded with 100 ng of purified protein. ERK8_KD sample (lane 6) is a point mutant on the conserved lysine (Lys, K) in position 42 to arginine (Arg, R). (D), Representative kinase assay blot of gatekeeper mutants (200 ng/sample of purified protein) (upper panel). Reactions were resolved by SDS-PAGE and ^32^P incorporation on MBP was estimated by densitometry. Coomassie staining verified that equal amounts of substrate were loaded (lower panel). Quantification of kinase activity in comparison to WT, as scored by MBP phosphorylation, from three independent experiments is reported in the lower panel.

The mechanism of action assessed by means of the previously described ATP competition assay was a solid indication that the selected molecules occupy the ATP cavity, as predicted. Moreover, being the demonstration of the binding mode particularly important to solidly validate our computational assumptions, we decided to deal with this issue also by an alternative approach. We took advantage of the so-called “chemical genetic analysis”, first performed by Shokat and co-workers [44] through the generation of engineered kinases sensitive to inhibitors and ATP analogues, which are not equally effective on wild type proteins. Indeed, a single residue in the ATP binding pocket of protein kinases, termed the “gatekeeper”, has been shown to control sensitivity to a wide range of small molecule inhibitors [45]–[47]. Therefore, we hypothesized that perturbing the ATP cavity of ERK8 by mutating its “gatekeeper” residue would affect the affinity of the ITT53 and ITT57 molecules only in case they bind to this specific region. This would confirm an ATP competitive mechanism of action for these hit compounds, as predicted by calculations and previously suggested with *in vitro* assays (Fig. 4B and Fig. 5B). Our analysis showed that the gatekeeper position of ERK8 (residue 92) is occupied by a phenylalanine (Phe, F) (Fig. 6A), differently from the templates used for the modeling (a glutamine for both FUS3 and ERK2) but similarly, for example, to members of the CDK family. Indeed, superimposition of our model to a high resolution CDK2 X-ray structure confirmed the local structural similarity in the gatekeeper surroundings between these two proteins (Fig. 6B), further supporting the reliability of our computational model. Based on this analysis and on previous studies of the gatekeeper position of CDK2 (CDK2_F80G) [48], [49], we decided to generate an ERK8 gatekeeper mutant substituting the Phe92 with a smaller glycine (Gly, G) residue. Unexpectedly, ERK8_F92G showed an almost complete loss in catalytic activity (Fig. 6D). Indeed, thanks to the broad use of the chemical genetic analysis, evidences about the ability of many kinases to tolerate dramatic mutations at the gatekeeper position have accumulated but, more recently, it has also become clear that many kinases do not tolerate such perturbations [50], [51]. These kinases (roughly 30% of tested kinases), that undergo loss of catalytic activity and/or cellular function upon introduction of space-creating gatekeeper mutations, have been classified as “intolerant”, unlike the “tolerant” ones that, in these conditions, are able to maintain their catalytic functions [51]. Although the selected structural templates FUS3 and ERK2, and the “gatekeeper-related” CDK2 protein all belong to the group of “tolerant” kinases, the ERK8_F92G loss of activity suggests that this protein could, therefore, represent a new “intolerant” kinase. In order to better investigate this aspect, we generated other three gatekeeper mutants substituting the phenylalanine with chemically different residues, namely another small amino acid, alanine (Ala, A), and two bulkier amino acids such as isoleucine (Ile, I) and tyrosine (Tyr, Y). All the generated single-point ERK8 mutants in the gatekeeper position, F92G, F92A, F92I, and F92Y, were expressed as N-terminal GST-tagged proteins in *E. coli* with the same procedure applied to the wild type protein. Purified fusion proteins were also controlled for correct identity via western blot analysis using a specific anti-ERK8 antibody, also demonstrating the expected molecular weight as compared to the wild type and kinase dead (ERK8_KD) proteins (Fig. 6C). Interestingly, all but the ERK8_F92I mutant showed a barely detectable basal activity in the kinase assay when it was measured in comparison to the wild type protein (Fig. 6D). These results support the classification of ERK8 as a new “intolerant” kinase to the mutation of the gatekeeper residue to glycine or alanine.

**Figure 7:**
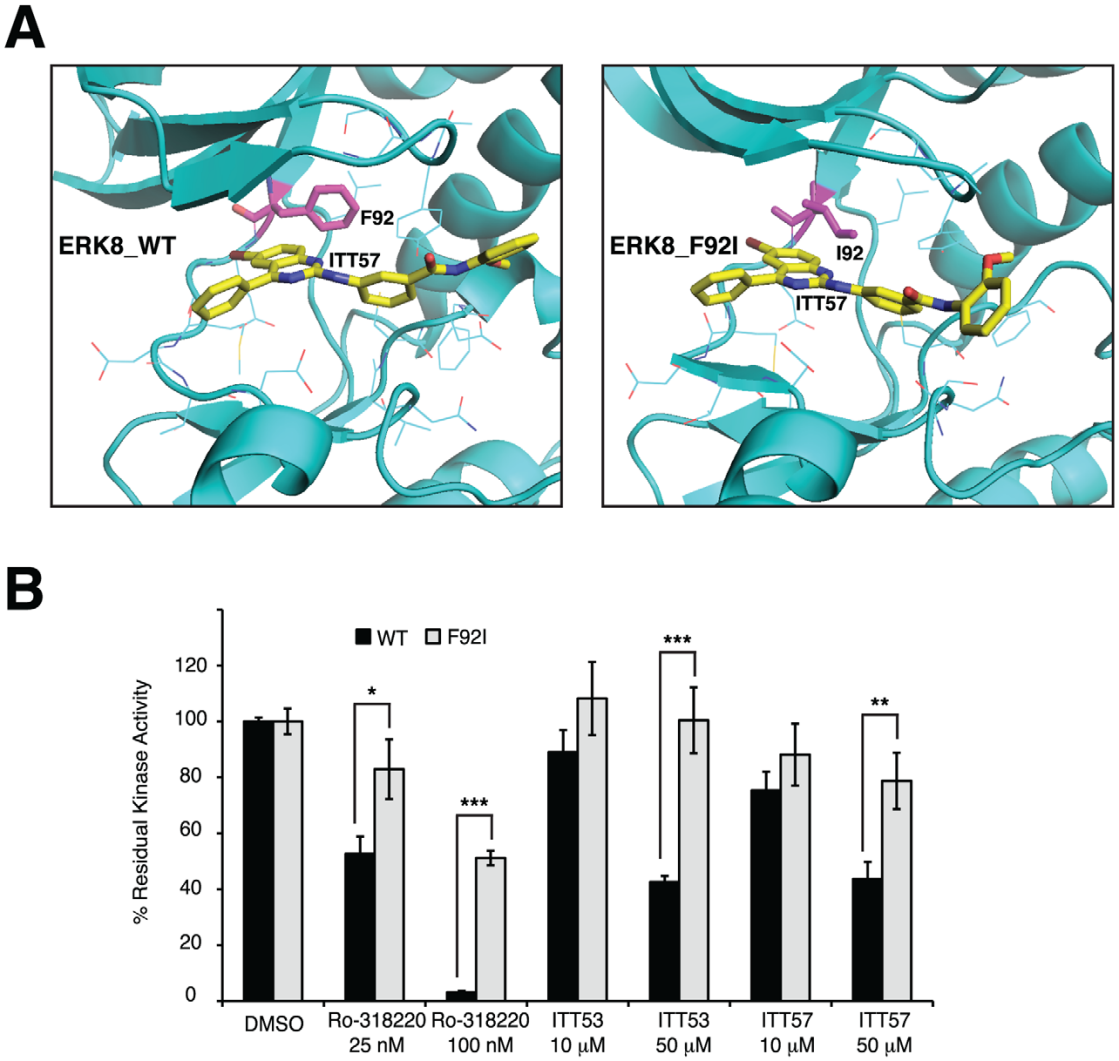
A resistant ERK8_F92I mutant confirms the predicted ATP pocket-binding mode. (A), Representative structures from MD simulation of the complex between ITT57 and both ERK8_WT (left panel) and ERK8_F92I mutant (right panel). The residue at position 92 is labeled and showed as sticks. The ITT57 ligand is showed as sticks. Protein residues and ligand atoms are colored by atom type. (B), GST tagged ERK8_WT and ERK8_F92I proteins (200 ng/sample) were used in kinase assays in presence of the indicated concentrations of ITT53, ITT57 and Ro-318220 molecules. Using the paper-spotted kinase assay technique, we quantified and normalized the activities of the WT and of the mutant protein. MBP phosphorylation levels were evaluated by β-counting protocol of triplicates and results expressed as percentage of residual kinase activity compared with control samples. Significance (p-value) was obtained by one-way ANOVA test. Asterisks were attributed for the following significance values: p<0.05 (*), p<0.01 (**), p<0.001 (***).

Being our goal the confirmation of the selected scaffolds binding mode and also the evaluation of the predictive value of ERK8 model, we next decided to focus our analysis only on the partially active ERK8_F92I mutant. Therefore, we generated the ERK8_F92I model with the same computational protocol applied to ERK8 wild type (WT). Then, we chose to analyze the interaction pattern of both modeled structures with one of our selected compounds. To this purpose, both ERK8_WT and ERK8_F92I, in complex with ITT57, were relaxed during 2 ns of unrestrained MD. The ITT57 delta energy of binding was calculated by means of the MMPBSA approach [52], showing that the substitution of the Phe92 aromatic ring with the aliphatic chain of isoleucine is associated to a higher delta energy of interaction. From a structural point of view, analysis of representative MD structures (Fig. 7A) revealed that the overall binding mode of ITT57 within the catalytic site is the same in both protein structures, although the methoxyphenyl moiety seems to be less buried into the lipophilic pocket of the ERK8_F92I mutant than into the wild type one. These differences, together with the possible enthalpy gain coming from a pi-pi stacking interaction between the Phe92 gatekeeper residue of ERK8_WT and ITT57, would suggest a less profitable interaction of ITT57 with the ERK8_F92I mutant than with the WT. Therefore, based on this *in silico* analysis, we could predict a decreased efficacy of ITT57 on the ERK8_F92I mutant. We next performed the conclusive experimental test to assess the binding mode of ITT53 and ITT57 by comparing ERK8_F92I and ERK8_WT inhibition profiles, this approach also giving us the possibility to challenge the theoretical prediction about ERK8_F92I increased resistance to ITT57. As a control, we also tested, in the same kinase assay, the Ro-318220 ATP competitive inhibitor [42]. As expected, we observed that the inhibitory activity of Ro-318220 was affected by the presence of the F92I mutation in ERK8, in particular being more active on the wild type than on the mutated ERK8 protein (Fig. 7B and Fig. S5). Similarly, ITT53 and ITT57 molecules were also significantly more active on ERK8_WT than on ERK8_F92I, proving that this kind of perturbation at the gatekeeper position gives rise to an ERK8 protein more resistant to inhibition triggered by small molecules that occupy the catalytic pocket. More importantly, this result both emphasizes the predictive ability of our model and confirms the competition assays data, ultimately showing that the selected scaffolds bind ERK8 in the ATP cavity.

## Discussion

Over the past decade, protein kinases raised as the pharmaceutical industry's most popular drug targets, especially in the field of cancer. In particular, interest in MAPKs has recently exploded [53], [54]. Also, chemical inhibitors, both the clinically relevant ones and many other molecules that do not reach the latest stages of approval procedures, have become invaluable reagents for studying the physiological functions of their target protein. However, the ATP binding sites with which most inhibitors interact, are highly conserved throughout the large kinase family, raising an acute specificity problem. Indeed, although this may sometimes be an advantage when it comes to clinical effect, most commercially available chemical inhibitors of protein kinases are poorly specific [28].

Knowledge about ERK8 targets and downstream effectors and, ultimately, about its biological functions is still limited. Among currently available data suggesting a role for this kinase in normal and aberrant cell proliferation [10], the recent observation about ERK8 being a potent regulator of telomerase activity [12] clearly shows the possibility of a beneficial use for pharmacological ERK8 inhibition. More specifically, it is shown that ERK8 pharmacological inhibition results in a significant decrease in telomerase activity, which tumor cells often activate to bypass replicative senescence and gain unlimited proliferation ability. In particular, Ro-318220 has been used to confirm the ERK8-dependent telomerase activity in transformed cell lines [12]. Altogether, these data strongly support the clinical potential of ERK8 inhibition.

The gap of knowledge to fill up about ERK8 signaling and the absence of a crystal structure led us to apply an *in silico* protocol to generate a 3D model of the ERK8 kinase domain and to screen, first *in silico* and then *in vitro*, molecular scaffolds to validate the ERK8 structure itself. As described above, whereas the structure-based derived subset showed a significant percentage (20%) of active molecules, the ligand-based approach only showed a 3% success rate. Although disappointing, such a low success rate indicated that the ERK8 inhibition observed with the other set of molecules was not a random effect based on an intrinsic high propensity of ERK8 for chemical inhibition.

Next, the structure-based model provided a valuable guidance in the screening of ATP competitive inhibitors, allowing us to identify a high percentage of *in vitro* active compounds (though with different effectiveness), despite the limited number of screened molecules. This result, together with the confirmation of their ability to target the ATP binding pocket, demonstrates that the generated ERK8 model is a reliable tool for the screening of novel inhibitors. These scaffolds, for the first time specifically selected towards the ERK8 ATP pocket, in turn can be worthy of further chemical optimization to increase and finely tune their potency.

Ultimately, this work also led us to the identification of an ERK8 drug-resistant mutant, namely ERK8_F92I, that not only proved the expected binding mode of our molecules, but could pave the way to the use of synthetic ATP analogues for the identification of ERK8 substrates [55]. Thanks to the study of gatekeeper mutations, we also demonstrated that space-creating mutations (i.e., glycine or alanine replacing the natural bulky phenylalanine) almost completely abolish kinase activity. Hence, we propose ERK8 as another member of the so-called “intolerant” group of kinases, as defined by Shokat and co-workers [51]. Altogether, our results suggest that the generated model will be an important resource for the identification of specific inhibitors for the ERK8 MAP kinase.

## Conclusions

A well-developed body of knowledge identifies different MAPKs as critical regulators of cell proliferation and human cancer. Several recently developed pharmacological inhibitors targeting MAPKs have been effective in animal models and have therefore advanced to clinical trials for the treatment of inflammatory diseases and cancer. Still, although the specific ERK8 member of this family has been proposed as a novel potential therapeutic target for cancer, the lack of its experimental structure currently limits the possibilities to efficiently look for pharmacological compounds specifically targeting this kinase. As a consequence, the development of new therapeutic strategies based on molecular and pharmacological intervention on ERK8 functions is currently impaired. We believe that our results show that the 3D ERK8 model we generated is a reliable tool to be exploited in a drug-design perspective. Consequently, future work about this atypical MAPK will largely benefit of the identification of a sufficiently specific inhibitor to dissect its signaling functions and to further validate its potential as a novel therapeutic target in cancer treatment.

## Supporting Information

Figure S1 **T-Coffee multiple sequence alignment.** Multiple sequence alignment between FUS3, ERK2 and ERK8 obtained with T-Coffee software (standard protocol). Consensus code: “yellow” indicates positions which have a single, fully conserved residue; “green” indicates conservation between groups of strongly similar properties; “blue” indicates conservation between groups of weakly similar properties. The TEY activation motif is in red.
(DOC)

Figure S2 **ERK8 model and FUS3.** (A), Superimposition of the ERK8 model (grey) with the FUS3 template (blue). (B), Superimposition of the refined ERK8 structure (cyan) with the FUS3 template (blue). (C), ADP binding mode within the catalytic pocket. In green sticks is showed the crystallographic binding mode of ADP within the FUS3 template. In grey sticks is showed the ADP binding mode in the ERK8 structure refined by molecular dynamics. In magenta sticks is showed ADP binding mode as obtained by self-docking ADP toward the refined ERK8 structure by the GOLD docking program. Heteroatoms are colored by atom types.
(DOC)

Figure S3 **Stability of MD.** Root mean square deviation (rmsd) of each frame with respect to the first frame of unrestrained MD, over time.
(DOC)

Figure S4 **Ligand-based approach: the training set.** List of compounds used to generate the two ligand-based pharmacophores (from Bain J, et al., 2007).
(DOC)

Figure S5 **Kinase Assay of GST-tagged ERK8 proteins on autophosphorylation.** (A), Representative kinase assay blot of WT and different ERK8 mutants (200 ng/sample of purified protein) (upper panel). Reactions were resolved by SDS-PAGE and ^32^P incorporation on GST-ERK8 proteins themselves was estimated by densitometry. Quantification of kinase activity in comparison to WT, as scored by autophosphorylation, from three independent experiments is reported in the lower panel. (B), GST tagged ERK8_WT and ERK8_F92I proteins (200 ng/sample) were used in kinase assays in presence of the indicated concentrations of ITT53, ITT57 and Ro-318220 molecules. Using the paper-spotted kinase assay technique, we quantified and normalized the activities of the WT and of the mutant protein. Autophosphorylation levels were evaluated by β-counting protocol of triplicates and results expressed as percentage of residual kinase activity compared with control samples.
(DOC)

Table S1 **Experimental Screening results for the structure-based selected molecules.** Ranking of all the molecules obtained with the structure-based approach: the percentage (in brackets) of residual kinase activity is reported for all the compounds. ^(a)^ residual kinase activity with respect to control samples containing no inhibitors ^(b)^ ratio between the number of active molecules and the number of tested molecules ^(c)^ success rate obtained for threshold activity up to 55% ^(d)^ success rate obtained for threshold activity up to 65%.
(DOC)
