# Supplementary figures and images for: Structure Prediction and Validation of the ERK8 Kinase Domain
Source: PLoS One. 2013 Jan 11;8(1):e52011. doi: 10.1371/journal.pone.0052011 (PMC3543423; doi:10.1371/journal.pone.0052011)

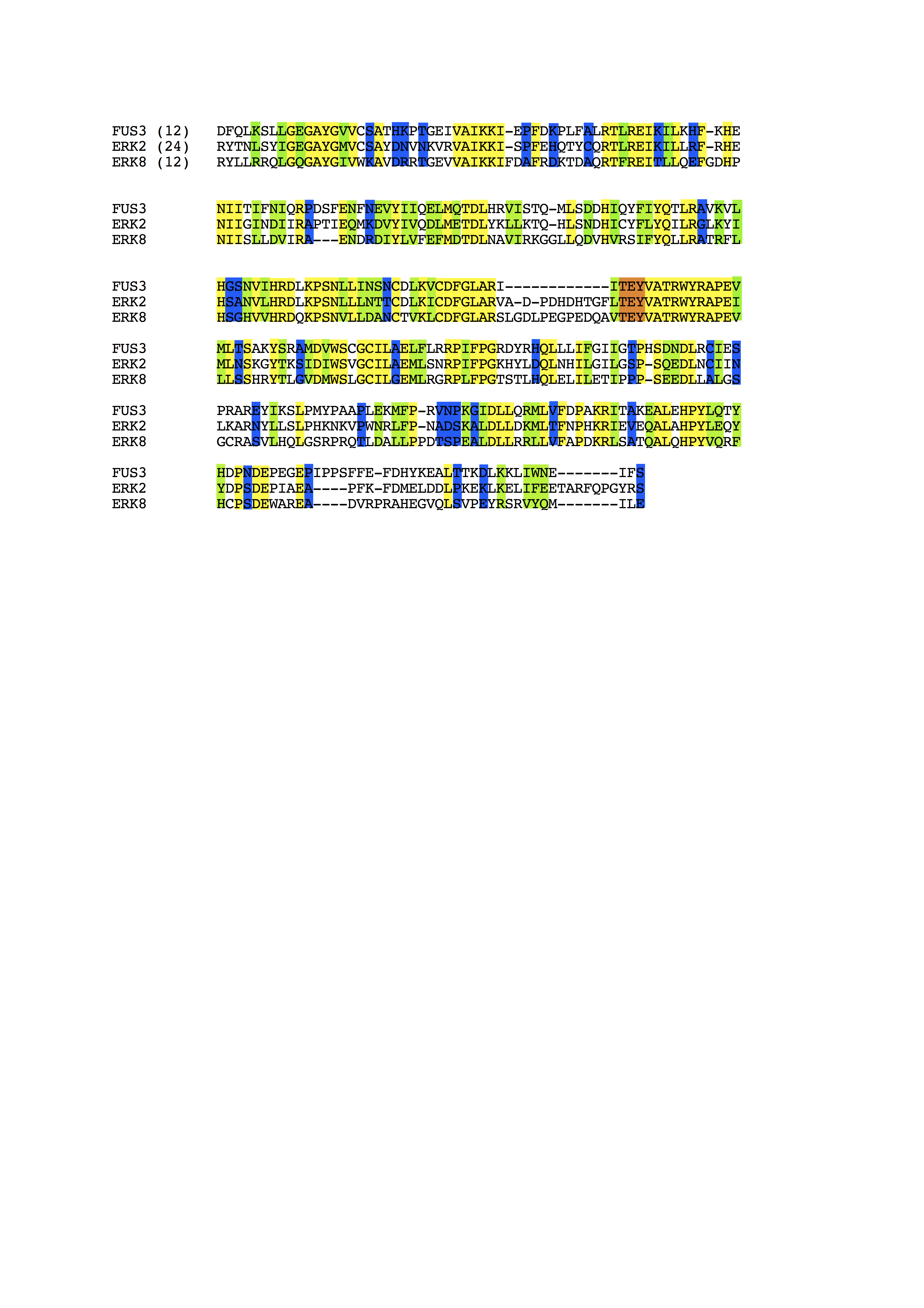

Supplement: Figure S1 — T-Coffee multiple sequence alignment. Multiple sequence alignment between FUS3, ERK2 and ERK8 obtained with T-Coffee software (standard protocol). Consensus code: “yellow” indicates positions which have a single, fully conserved residue; “green” indicates conservation between groups of strongly similar properties; “blue” indicates conservation between groups of weakly similar properties. The TEY activation motif is in red. (DOC) [file pone.0052011.s001.doc]

**
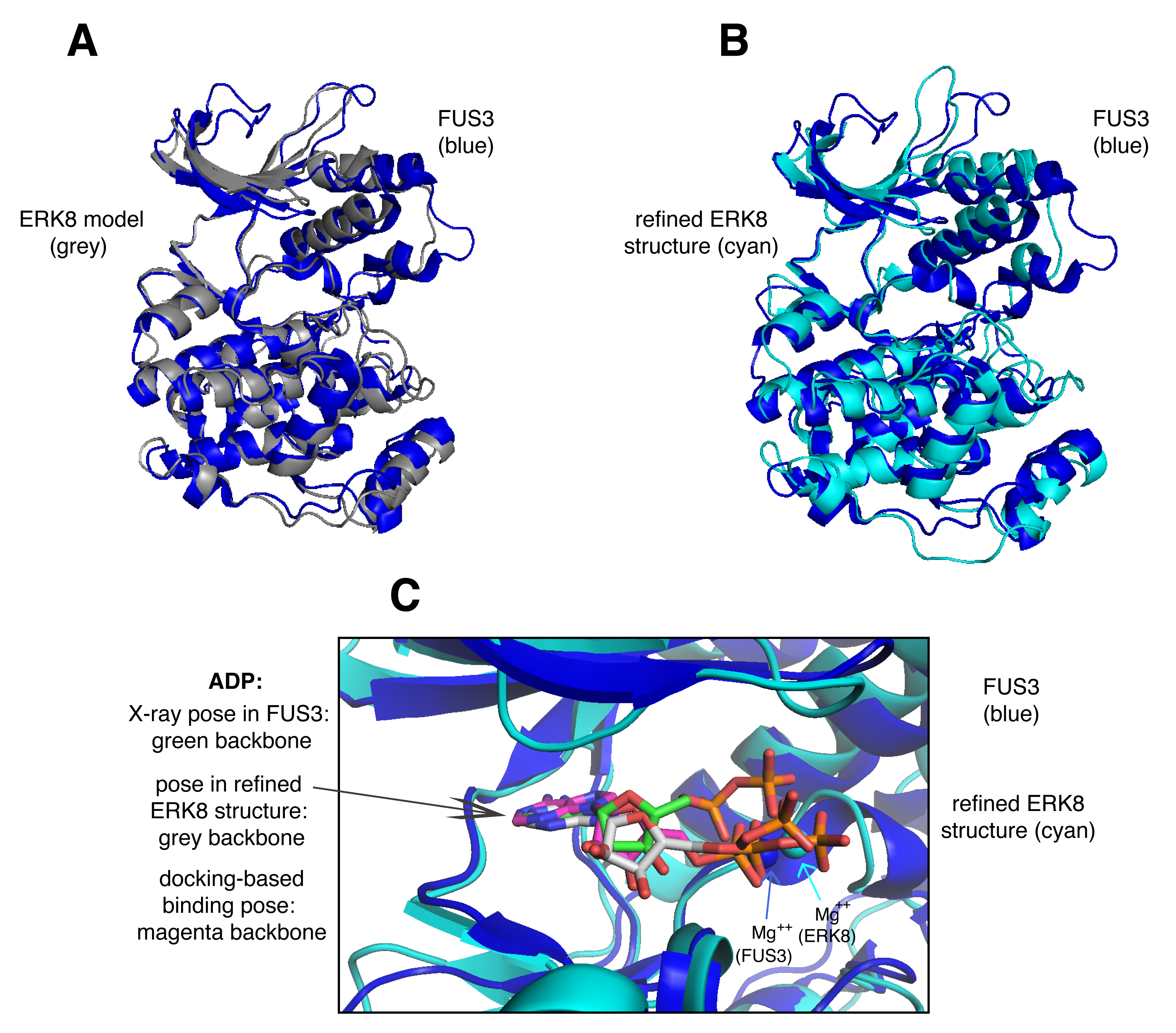
**

Supplement: Figure S2 — ERK8 model and FUS3. (A), Superimposition of the ERK8 model (grey) with the FUS3 template (blue). (B), Superimposition of the refined ERK8 structure (cyan) with the FUS3 template (blue). (C), ADP binding mode within the catalytic pocket. In green sticks is showed the crystallographic binding mode of ADP within the FUS3 template. In grey sticks is showed the ADP binding mode in the ERK8 structure refined by molecular dynamics. In magenta sticks is showed ADP binding mode as obtained by self-docking ADP toward the refined ERK8 structure by the GOLD docking program. Heteroatoms are colored by atom types. (DOC) [file pone.0052011.s002.doc]

**
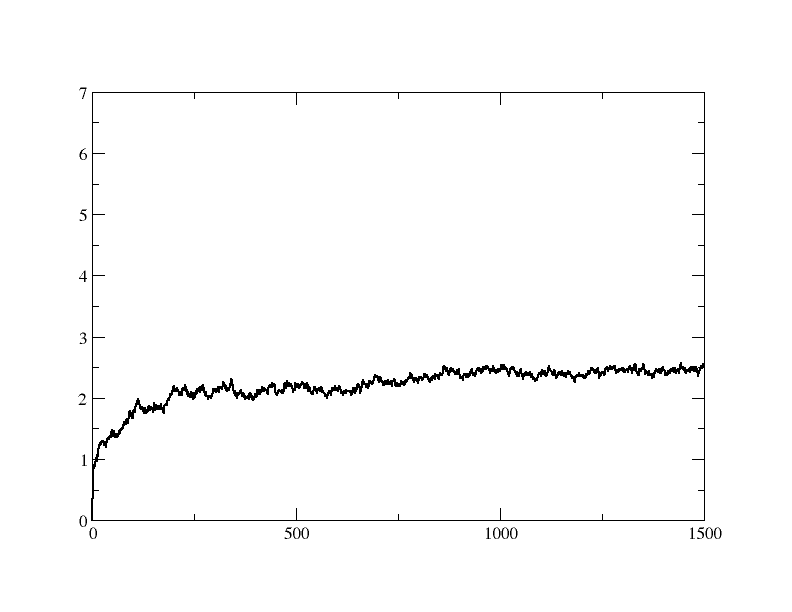
**

rmsd (Å)

time (ps)

Supplement: Figure S3 — Stability of MD. Root mean square deviation (rmsd) of each frame with respect to the first frame of unrestrained MD, over time. (DOC) [file pone.0052011.s003.doc]

**
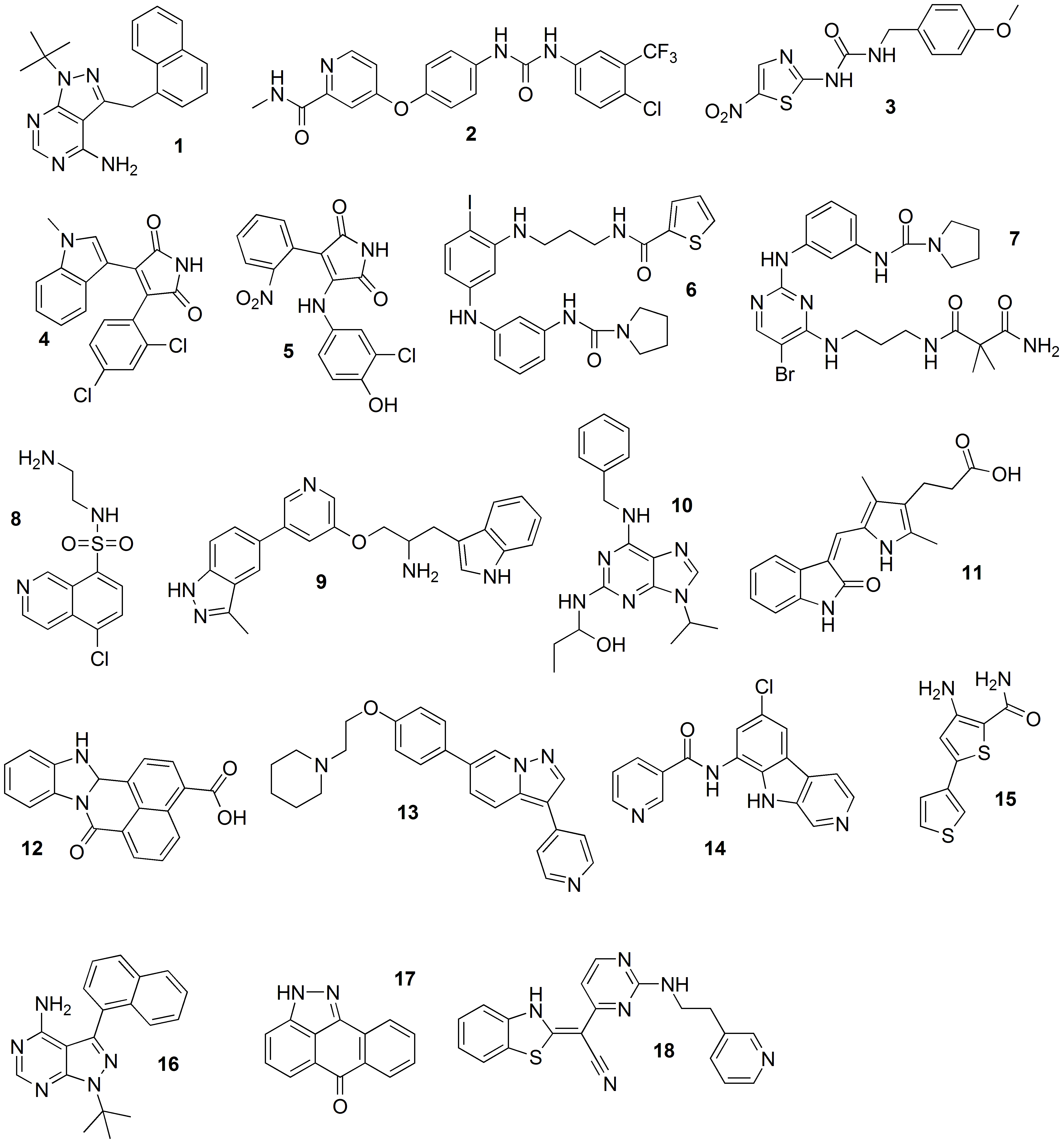
**

Supplement: Figure S4 — Ligand-based approach: the training set. List of compounds used to generate the two ligand-based pharmacophores (from Bain J, et al., 2007). (DOC) [file pone.0052011.s004.doc]

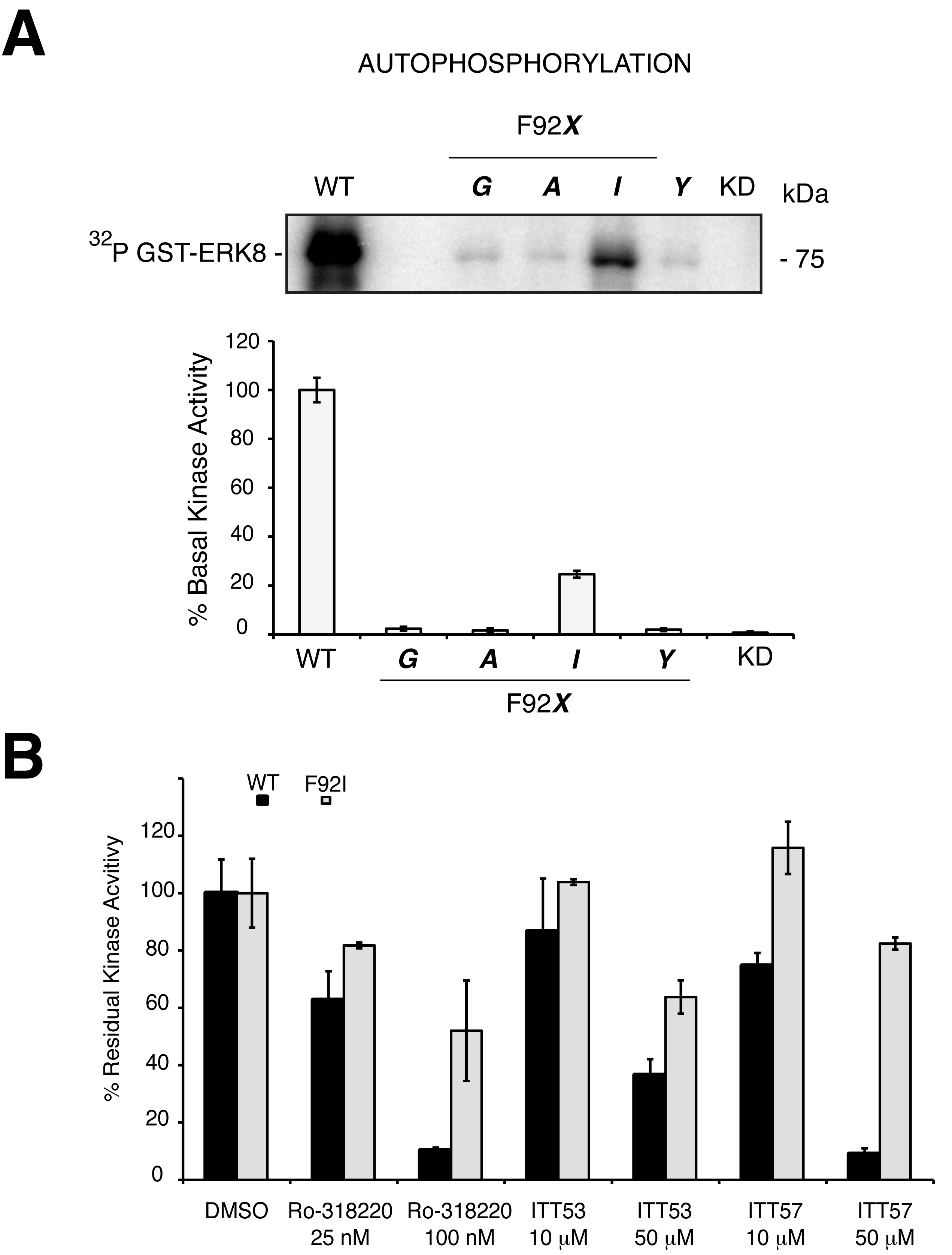

Supplement: Figure S5 — Kinase Assay of GST-tagged ERK8 proteins on autophosphorylation. (A), Representative kinase assay blot of WT and different ERK8 mutants (200 ng/sample of purified protein) (upper panel). Reactions were resolved by SDS-PAGE and 32P incorporation on GST-ERK8 proteins themselves was estimated by densitometry. Quantification of kinase activity in comparison to WT, as scored by autophosphorylation, from three independent experiments is reported in the lower panel. (B), GST tagged ERK8_WT and ERK8_F92I proteins (200 ng/sample) were used in kinase assays in presence of the indicated concentrations of ITT53, ITT57 and Ro-318220 molecules. Using the paper-spotted kinase assay technique, we quantified and normalized the activities of the WT and of the mutant protein. Autophosphorylation levels were evaluated by β-counting protocol of triplicates and results expressed as percentage of residual kinase activity compared with control samples. (DOC) [file pone.0052011.s005.doc]

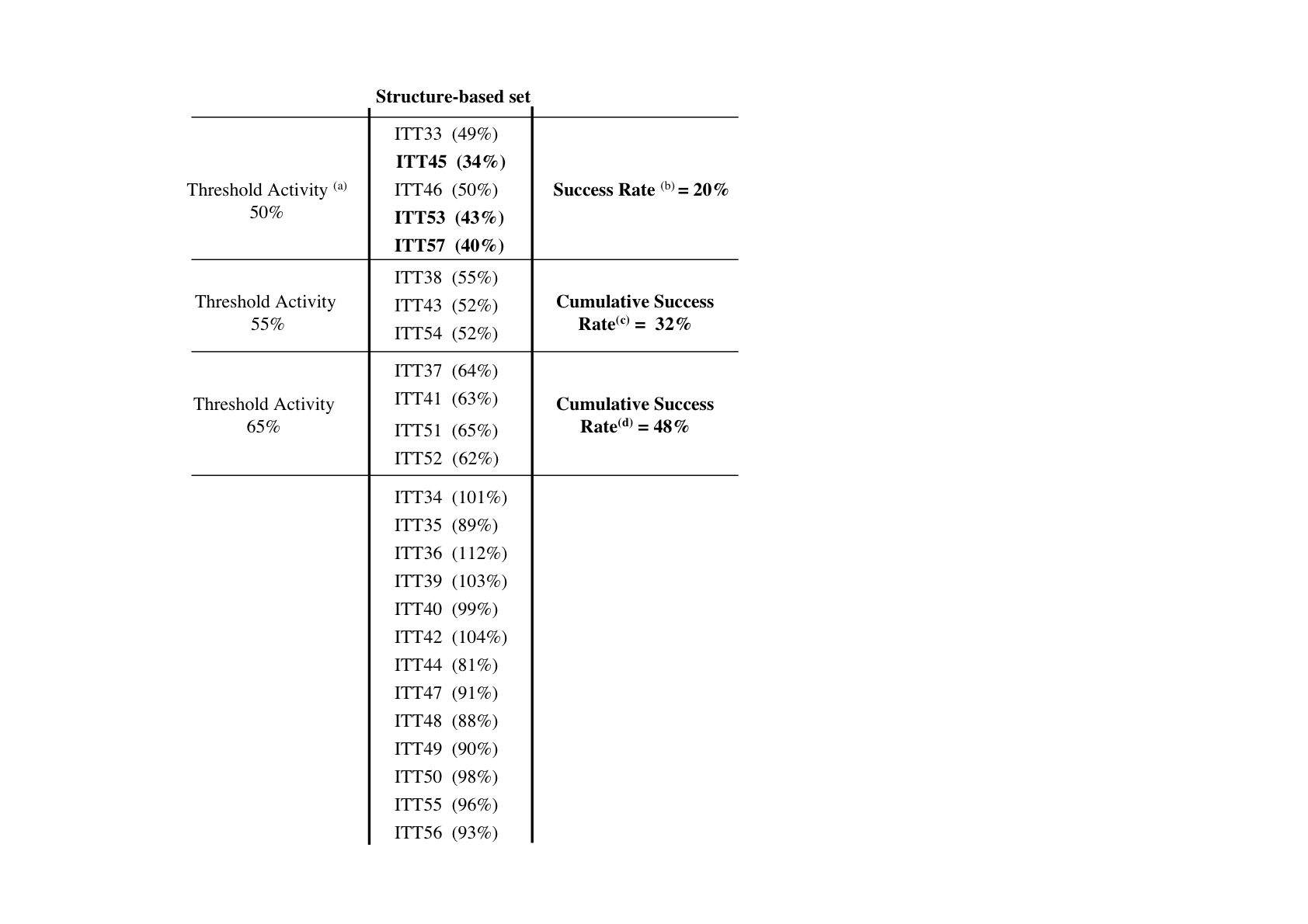

Supplement: Table S1 — Experimental Screening results for the structure-based selected molecules. Ranking of all the molecules obtained with the structure-based approach: the percentage (in brackets) of residual kinase activity is reported for all the compounds. (a) residual kinase activity with respect to control samples containing no inhibitors (b) ratio between the number of active molecules and the number of tested molecules (c) success rate obtained for threshold activity up to 55% (d) success rate obtained for threshold activity up to 65%. (DOC) [file pone.0052011.s006.doc]
